# Letter from Dr. Herrick

**Published:** 1846-12

**Authors:** 


### ARTICLE XIV.

### LETTER FROM DR. HERRICK.

We have received a letter from our colleague, who was appointed to the Medical Staff of the army that has marched against Mexico, dated "Banks of the Elhonda, 56 miles from San Antonio, Texas, Oct. 5th, 1846," he being surgeon to the 1st regiment of Illinois volunteers, under command of General Hardin, which with the 2d regiment had joined the army under the command of General Wool, and were on their march to Mexico.

There has been considerable sickness in this detachment of our army. He says: "They have been afflicted with measles, mumps, and numerous other diseases, besides those peculiar to the climate; yet after all the mortality has not been great, not more than seven men having died out of the 1st regiment.

The letter closes by saying that we may, in a few days, expect an article from him for the Journal.

